# SPI-23 of *S*. Derby: Role in Adherence and Invasion of Porcine Tissues

**DOI:** 10.1371/journal.pone.0107857

**Published:** 2014-09-19

**Authors:** Matthew R. Hayward, Manal AbuOun, Roberto M. La Ragione, Monika A. Tchórzewska, William A. Cooley, David J. Everest, Liljana Petrovska, Vincent A. A. Jansen, Martin J. Woodward

**Affiliations:** 1 Department of Structural and Computational Biology, European Molecular Biology Laboratory, Heidelberg, Germany; 2 Department of Bacteriology, Animal Health and Veterinary Laboratories Agency, Surrey, United Kingdom; 3 School of Biological Sciences, Royal Holloway University of London, Surrey, United Kingdom; 4 School of Veterinary Medicine, Faculty of Health and Medical Sciences, University of Surrey, Surrey, United Kingdom; 5 School of Veterinary Sciences, University of Bristol, Bristol, United Kingdom; 6 Microscopy Facility, Animal Health and Veterinary Laboratories Agency, Surrey, United Kingdom; 7 Department of Food and Nutritional Sciences, University of Reading, Reading, United Kingdom; The Biodesign Institute, Arizona State University, United States of America

## Abstract

*Salmonella enterica* serovars Derby and Mbandaka are isolated from different groups of livestock species in the UK. *S*. Derby is predominantly isolated from pigs and turkeys and *S*. Mbandaka is predominantly isolated from cattle and chickens. Alignment of the genome sequences of two isolates of each serovar led to the discovery of a new putative *Salmonella* pathogenicity island, SPI-23, in the chromosome sequence of *S*. Derby isolates. SPI-23 is 37 kb in length and contains 42 ORFs, ten of which are putative type III effector proteins. In this study we use porcine jejunum derived cell line IPEC-J2 and *in vitro* organ culture of porcine jejunum and colon, to characterise the association and invasion rates of *S.* Derby and *S*. Mbandaka, and tissue tropism of *S*. Derby respectively. We show that *S*. Derby invades and associates to an IPEC-J2 monolayer in significantly greater numbers than *S*. Mbandaka, and that *S*. Derby preferentially attaches to porcine jejunum over colon explants. We also show that nine genes across SPI-23 are up-regulated to a greater degree in the jejunum compared to the colon explants. Furthermore, we constructed a mutant of the highly up-regulated, *pilV*-like gene, *potR*, and find that it produces an excess of surface pili compared to the parent strain which form a strong agglutinating phenotype interfering with association and invasion of IPEC-J2 monolayers. We suggest that *potR* may play a role in tissue tropism.

## Introduction

*Salmonella enterica* is an important zoonotic pathogen of warm blooded vertebrates, including humans and livestock. The symptoms of Salmonellosis include chronic gastroenteritis, affecting a wide range of host species and caused primarily by broad host range serovars, and an often fatal typhoid fever affecting a narrow range of host species, caused primarily by host limited or restricted serovars [1]–[3]. International serotyping has provided statistics that have elucidated links between certain serovars and a defined host species range [4]–[7].

In previous work the chromosome sequences of *S. enterica* serovars, Derby and Mbandaka were compared [8]. Isolation statistics suggest that these serovars have different host species biases in the UK: *S*. Derby is prominently isolated from pigs (40%) and turkeys (50%) and *S*. Mbandaka from chickens (65%) and cattle (20%) [5], [8]. Alignment of the chromosome sequences led to the discovery of a new putative *Salmonella* pathogenicity island (SPI) in isolates of *S*. Derby, designated SPI-23 [8]. SPI-23 is 37 kb in length and encodes 42 genes, ten of which were identified by the online tool SEIVE as putative type 3 effector proteins; of these eight were unique in nucleotide sequence to *S*. Derby [8], [9]. Type III effector proteins are important pathogenicity factors secreted through the type III secretion system in to a host cell where they modulate cell signalling, in some instances pacifying the hosts immune system or aiding in invasion of, or translocation across, the intestinal epithelial barrier [10], [11].

Considering the high number of putative pathogenicity genes on SPI-23 and the higher number of isolations of *S*. Derby from pigs in the UK it was posited that this island may play a role in pathogenicity in this host [8]. In the current study we undertake preliminary studies to start addressing this hypothesis; we show that *S*. Derby associates to, and invades, a porcine derived monolayer faster than *S.* Mbandaka and that SPI-23 is regulated in a tissue specific fashion. Furthermore a knock-out mutant of the most up-regulated gene, *potR*, results in cellular agglutination in a static culture. We discuss the possible role that *potR* and other SPI-23 genes may play in tissue tropism.

## Results and Discussion

### *S*. Derby associates and invades IPEC-J2 monolayers in significantly greater numbers than *S*. Mbandaka and shows a preference to associating with jejunum over colon explants

*S*. Derby is frequently isolated from pigs, while *S.* Mbandaka is rarely isolated from pigs in the UK [5]–[7]. To advance beyond the inferences made from comparative functional genomics regarding host adaptations we decided to see if *S*. Derby was more proficient in associating (adhering and invading) with, and invading, a porcine jejunum derived cell line (IPEC-J2) than *S*. Mbandaka. IPEC-J2 monolayers have been used previously as a model for the porcine jejunum, a site associated with the invasion of *Salmonella enterica* [12]–[14]. The rate of invasion and association was studied through varying lengths of incubation of *S*. Derby and *S*. Mbandaka isolates with IPEC-J2 monolayers.

**Figure 1:**
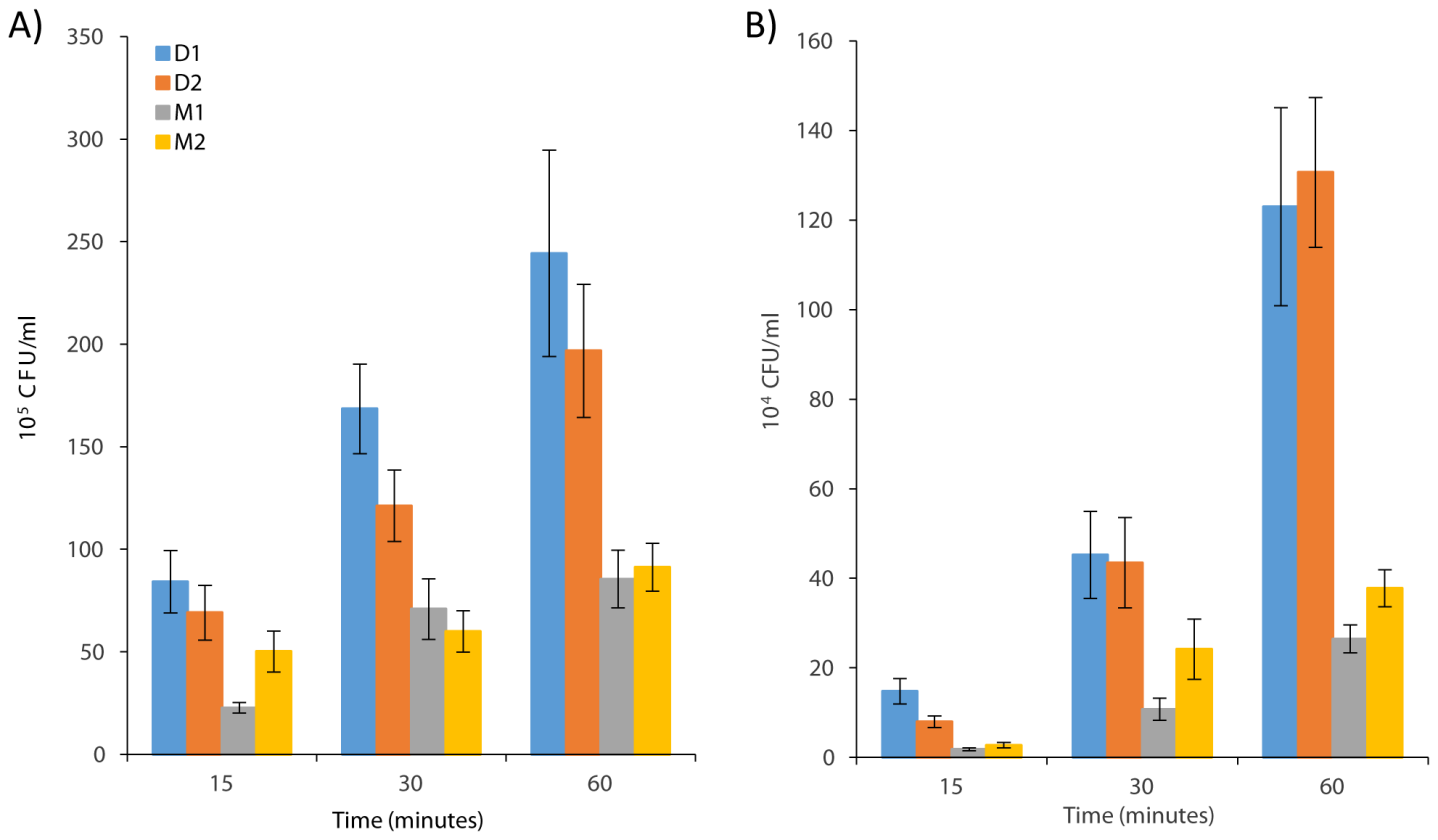
Association and invasion of IPEC-J2 monolayers by *S*. Derby and *S*. Mbandaka. (**a**) Association (cells adhering and invading) and (**b**) invasion of IPEC-J2, porcine jejunum derived cell line, by *S*. Derby strains D1 and D2 and *S*. Mbandaka strains M1 and M2 after 15, 30 and 60 minute incubation periods. Values are mean colony forming units (CFU) +/−1SEM recovered from disrupted monolayers planted on to LB agar plates and incubated for 16 hours at 37°C.

From 15 minutes onwards there were significantly more *S*. Derby associated to the monolayer than there were *S*. Mbandaka (p<0.05), with the exception of *S.* Derby D1 and *S.* Mbandaka M2 at 15 minutes, which were not significantly different (p>0.05) (**Figure 1**). There was a significantly greater number (p<0.05) of *S*. Derby inside IPEC-J2 cells than *S.* Mbandaka at all-time points, with the exception of *S*. Derby D2 and *S*. Mbandaka M2 at 30 minutes, which were not significantly different (p>0.05). After 60 minutes there were approximately 2.5 times as many *S*. Derby cells associated with the monolayer and approximately 4 times as many cells internalised than *S*. Mbandaka cells. Control strain *E. coli* K12 was not recovered after gentamicin treatment indicating that this step was effective in killing the Gram negative bacteria on the surface of the monolayer.

Though other studies have found poor concordance between monolayer invasion rates, pathogenicity and host association, the use of monolayers here shows that *S*. Derby is more proficient at associating and invading IPEC-J2 monolayers than *S*. Mbandaka [15], [16]. It was previously shown, with two strains of *S*. Typhimurium, that differences in invasion rates observed in IPEC-J2 assays were comparable to those found in porcine mucosal explants [17]. Though we may not assert that the more proficient association and invasion by *S*. Derby is the determinant of the bias towards a porcine host, we can suggest that *S*. Derby possesses adaptations for aspects of pathogenesis of the porcine host, that are absent in the comparator serovar, *S*. Mbandaka. Of course, it would be interesting to undertake a broader study of populations of a variety of serovars, especially the promiscuous serotypes such as *S*. Typhimurium, to assess whether this finding holds true across different serovars.

The colon is also a site commonly associated with *Salmonella* invasion [18], [19]. To assess if there was preferential attachment by *S*. Derby to either of these tissues, porcine IVOC association assays were performed. Both *S*. Derby strains associated in significantly greater numbers with jejunum when compared to colon (p<0.05) (data not shown). After 30 minutes there were 2.5 times as many *S*. Derby D1 cells associated with the jejunum than the colon. For strain *S*. Derby D2 there were 1.5 times as many cells associated to jejunum than colon tissues. This suggests that *S*. Derby preferentially attaches to the jejunum over the colon. No colonies were recovered from the PBS controls, suggesting that colonies observed in the IVOC association assays were not contaminates from residual gut microbiota.

### *Salmonella* pathogenicity island 23 (SPI-23) is up-regulated in the porcine jejunum IVOC preparations

In light of the preferential attachment of *S*. Derby to porcine jejunum compared to colon and the need to characterise the newly discovered SPI-23 in relation to pathogenesis in the porcine host, we investigated if there was a difference in transcriptional regulation of the island when the serovar was exposed to the different tissue types. Due to the greater fold difference between the number of *S*. Derby D1 cells associated with the jejunum and colon tissues compared to that of *S*. Derby D2, this strain was selected to study the potential differences in the expression of putative pathogenicity island, SPI-23, when exposed to jejunum and colon tissue explants.

**Figure 2:**
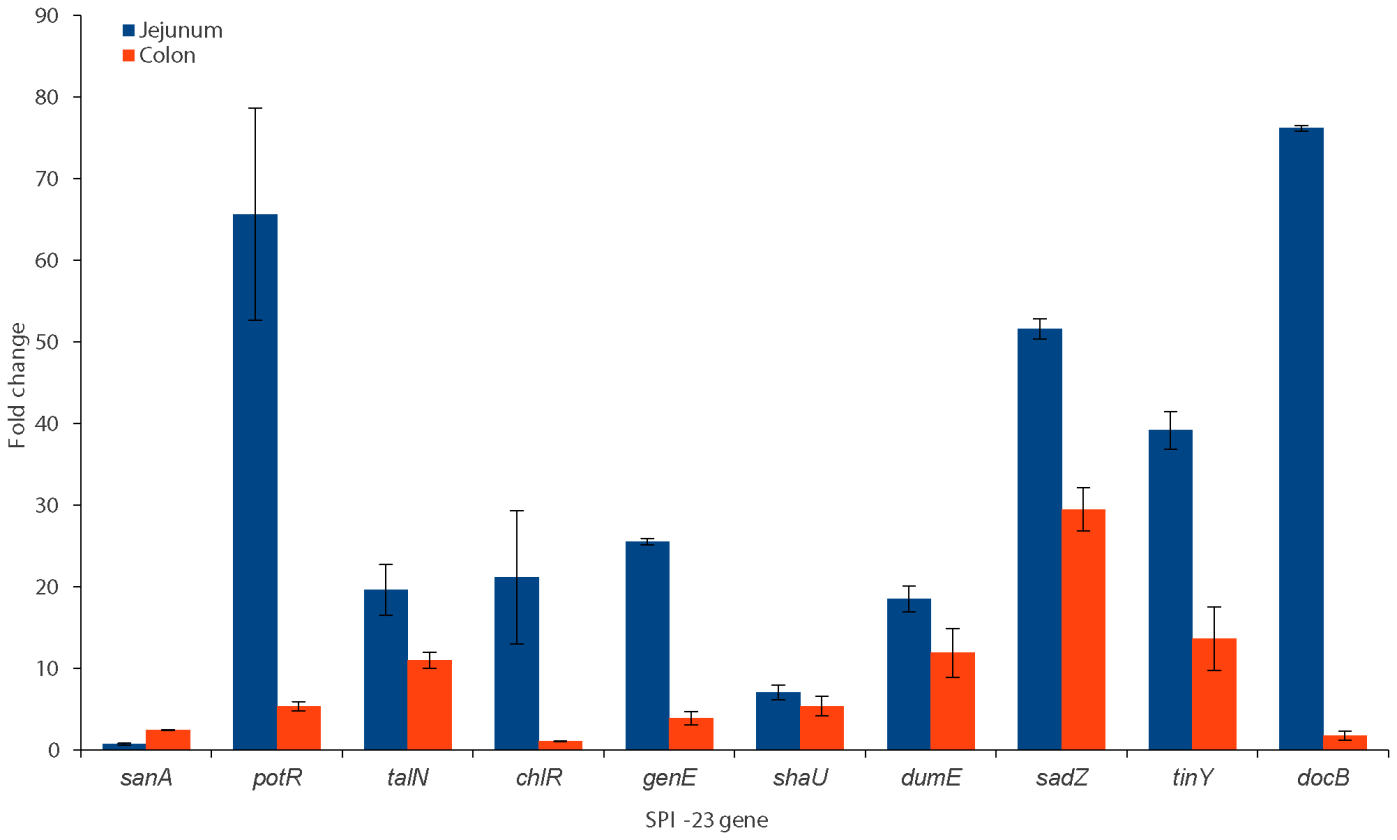
Expression of SPI-23 in *S*. Derby D1 when exposed to jejunum and colon explants. Fold differences in qRT-PCR expression levels +/−1SEM of ten genes found on SPI-23, in the order they appear on the island, when exposed to porcine jejunum and colon explants relative to a no tissue control.

The gene *potR* and the putative type three effector protein genes *genE*, *sadZ*, *tinY* and *docB* were up-regulated to a significantly greater degree (p<0.05) in jejunum when compared to colon, with fold changes from the no tissue control of between 21.6 and 74.4 (**Figure 2**). The putative type III effector protein, *sanA*, was the only gene expressed to a significantly greater degree in colon than in jejunum explants (p<0.05). The fold differences in expression levels from no tissue controls of the putative pilin protein gene *talN* and the putative type III effector protein genes *chlR*, *shaU* and *dumE* were not significantly different between the jejunum and colon explants (p>0.05). The largest significant fold change was observed for the gene *docB*, a putative type III effector protein, which was up-regulated 74.4 times more in the jejunum than in the colon. It was previously shown that 17 genes were unique to SPI-23 of *S*. Derby, whereas the gene *docB* was shown to be highly conserved in SPI-23 of *S*. Agona, *S*. Dublin and *S*. Gallinarum [8]; this raises the question about its expression and role in these host bacteria.

The *pilV-*like gene *potR,* previously shown to be unique in amino acid sequence to the SPI-23 of *S*. Derby D1 and D2, has the second largest difference between tissues, with a 13 fold greater expression level in the jejunum compared to colon [8]. The smallest significant difference between fold change from the no tissue control between the jejunum and colon treated cells was for the gene *sanA,* with a 3.5 fold greater level of transcription in colon samples than in jejunum. The up regulation of a subset of SPI-23 genes in the jejunum compared to both the colon and no tissue control suggests that the island may play a part in the preferential adherence and invasion in IVOC assays in jejunum rather than colon tissue.

### Sequence features of *potR* and the phenotype of *S.* Derby D1 *ΔpotR::kan*

Due to the novelty of the gene *potR* in the SPI-23 of *S*. Derby, the high degree of up-regulation of the gene during exposure to jejunal tissue explants and the preferential association of the isolate to jejunal tissue explants, we characterised the phenotype of this gene further.

As a preliminary step to determine the role of *potR* in *S*. Derby we identified the conserved protein domains. *potR* encodes 495 amino acid residues, sharing 89% with the putative *pilV*-like partial protein ZP_12137808 from the genome sequence of *Salmonella* Hvittingfoss strain A4-620. BLASTp showed the first 405 amino acids of *potR* consist of a multi-domain region containing a shufflon domain (5-405aa, e-value 6.22e^-17^), a *pulG* pseudopilin motif domain (1-134aa, e-value 9.65e^-04^), a *gspG* type II export sequence motif domain (5-56aa, e-value 8.88e^-03^) and a type IV pilin methylation domain (1-26aa, e-value 3.83e^-03^). These domains are consistent with other putative *pilV* genes, including *Salmonella* Hvittingfoss strain A4-620 and *Salmonella* Typhi strain CT18 (BLASTP performed 3/4/14). The region between amino acids 405 and 495 had no identifiable conserved domain. In *S*. Typhi the PilV protein is located to the tip of the self-aggregating pilus, attaching to a larger PilS protein, causing the pili to detach from the cell surface in a particular conformation of the shufflon, allowing cells to remain planktonic [20].

**Figure 3:**
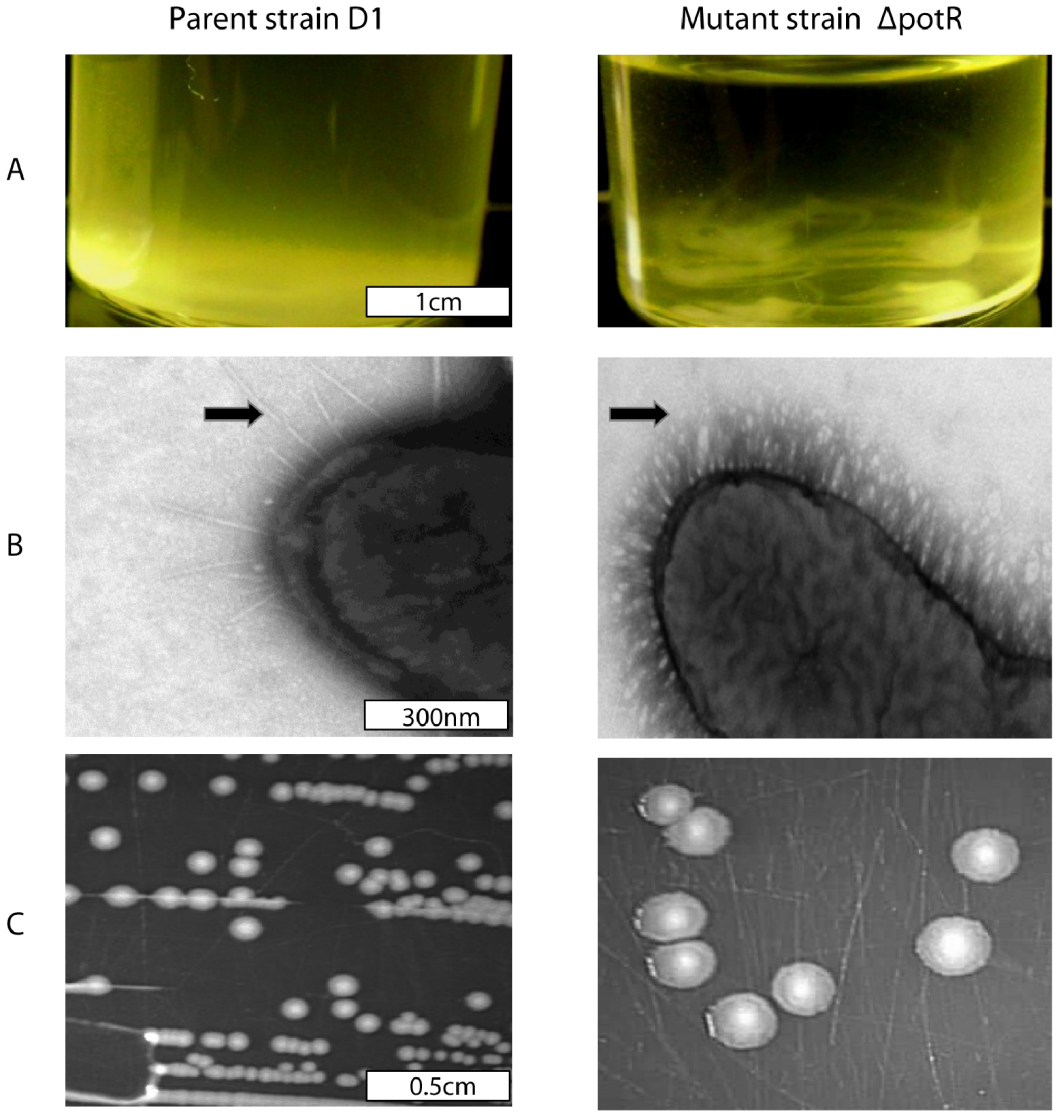
Comparison of morphological and structural features of the *S*. Derby D1 parental and *potR* mutant strains. Comparison of *S*. Derby D1 (left) and mutant strain *S*. Derby D1 *ΔpotR::kan* (right). (**a**) Strains were photographed after 2 hours remaining static post 16 hours of culturing at 37°C with agitation at 220 rpm. (**b**) Negative electron microscopy performed after 16 hours of culturing at 37°C with agitation at 220 rpm. Pili on the mutant and type-1 fimbriae on the parental strain are marked by arrows. (**c**) Strains were plated on to LB agar plates and incubated for 16 hours at 37°C.

Allelic exchange mutagenesis with a Kanamycin cassette produced the strain *S*. Derby D1 *ΔpotR*::kan, which displayed a strong agglutinating phenotype when left statically at room temperature (**Figure 3a**) suggesting that *potR* is isofunctional to *pilV* [20]. We were unable to remove the kanamycin cassette from *S*. Derby D1 *ΔpotR*::kan and complement *potR* despite many attempts at electroporation this is presumably due to the strong agglutination of a static culture.

Negative stain microscopy of overnight planktonic cultures of *S*. Derby D1 and *S*. Derby D1 *ΔpotR::kan* showed clearly that the mutant strain displayed a much higher number of pili on its cell surface when compared to the parent strain (**Figure 3b**). This suggests that either pili were upregulated or unable to dissociate from the cell surface. Colony morphology also distinguished the mutant from the parent strain. Plating planktonic cultures of parent and mutant strains onto LB agar plates resulted in the formation of fewer and larger colonies by the mutant strain (**Figure 3c**). Both parent and mutant colonies were of smooth morphology. Yet half of the diameter skirting the outer side of the mutant colonies was translucent, this was absent from the parent colonies which were opaque to the margins.

**Figure 4:**
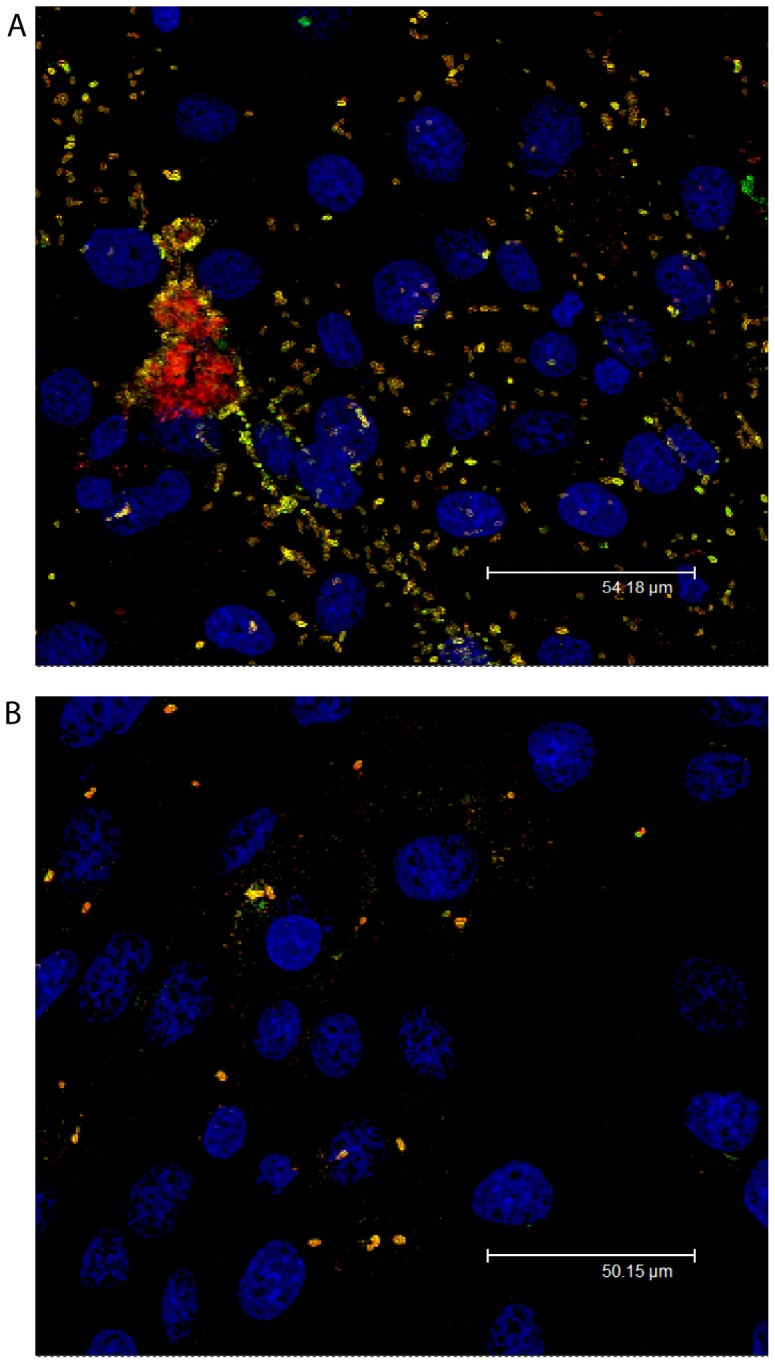
Adhesion and Invasion of *S*. Derby D1 parent and *potR* mutant strains. Differential stain confocal microscopy of (**a**) *S*. Derby D1 and (**b**) *S*. Derby D1 *ΔpotR::kan* that have adhered (yellow) and invaded (red) IPEC-J2 monolayers (blue) after 4 hours exposure.

To evaluate the effect of the *ΔpotR* phenotype on the interaction between *S*. Derby and the porcine jejunum, confocal microscopy was performed on IPEC-J2 monolayers after 4 hours of exposure to planktonic mid-log cultures of *S*. Derby D1 and *S*. Derby D1 *ΔpotR*::kan. Inspection of confocal preparations showed that the parent strain adhered in much greater numbers than the mutant (**Figure 4**). Hence, it may be inferred that the agglutinating phenotype hampered the ability of Derby D1 *ΔpotR*::kan to adhere to and invade IPEC-J2 monolayers. It was suggested by Morris et al. (2003) that the aggregating phenotype seen with the down regulation of *pilV,* found on SPI-7 of *S*. Typhi, may increase the number of cells invading the small intestine in humans [21], [22]. They proposed that internalisation of aggregated cell masses would increase the intracellular load of the pathogen compared to single cell invasion events, though they found no evidence of mutation in *pilV* affecting the number of cells invading INT407 human cells [21]. Here we show that the *pilV*-like gene, *potR* knock out produces a highly self-aggregating phenotype that leads to lower numbers of cells adhering and fewer cells invading the porcine jejunal monolayer. This would suggest the converse to the hypothesis put forward by Morris et al. (2003), namely that the bacteria are likely to be planktonic in the lumen of the porcine jejunum, where *S*. Derby preferentially associates and where *potR* is upregulated, which potentially allows a greater degree of adhesion and invasion, as cells can cover a larger surface area, through the formation of detachable, self-aggregating pili [21]. A similar hypothesis has been proposed for an aggregative strain of *S.* Typhimurium which is less pathogenic than a non-aggregating strain in a mouse model [22].

## Conclusions

We have shown (a) increased association to porcine jejunum derived monolayers of *S*. Derby (commonly isolated from pigs) compared to *S*. Mbandaka (rarely isolated from pigs) for which we previously performed in depth comparative functional genomics (b) the preferential association of *S.* Derby to porcine jejunum explants and (c) the greater up-regulation of SPI-23 genes in the jejunum compared to the colon explants that collectively may be taken as supportive evidence to indicate that SPI-23 contributes to tissue tropism. This hypothesis is further supported here by the characterisation of the gene *potR.* We have shown that a *S*. Derby knock-out mutant of the *pilV* –like gene *potR* is unable to adhere to or invade porcine jejunum derived monolayers. We speculate that this phenotype may be linked to the observed tissue tropism, as the high expression level of *potR* in the jejunum may allow the culture to remain planktonic and therefore invade a larger surface area compared to the colon where *potR* expression is lower. Further experiments are required to test these hypotheses namely inoculation studies in a porcine model.

## Methods and Materials

### Strains and culturing

The strains used in this study, *S*. Derby D1 and D2 and *S*. Mbandaka M1 and M2, were selected as their chromosome sequences have been previously annotated and compared [8]. The strains were isolated from different geographical locations during background monitoring performed on livestock in the UK between 2000 and 2010. *S*. Derby isolates D1 and D2 were obtained from pigs in 2008 and *S.* Mbandaka isolates M1 and M2 were obtained in 2008 and 2009 from cattle [8].

Unless stated otherwise, the strains were grown at 37°C for 16 hours aerobically either on LB agar plates or in LB broth agitated vigorously at 220 rpm. For porcine IVOC and IPEC-J2 assays mid-log cultures were used; this was achieved through inoculation of 20 ml of pre-warmed LB with 200 µl of a 16 hour overnight culture, followed by incubation for 3 hours at 37°C with agitation at 220 rpm. A frozen stock cultures was made of the *potR* mutant, *S*. Derby D1 *ΔpotR*::kan directly from a planktonic culture. Due to the strong agglutinating phenotype beads were made directly from fresh planktonic cultures grown for 16 hours in LB broth at 37°C with agitation at 220 rpm. From the culture, 100 µl of planktonic culture was mixed in a 2 ml CryoVial (Fisher Scientific, USA) containing 1.5 ml of HIB (brain heart infusion broth) + 30% glycerol and quickly placed into a −80°C freezer, to freeze the culture in its planktonic state.

### Culturing and infection of IPEC-J2 monolayers

Association and invasion assays using the porcine jejunum derived IPEC-J2 monolayers were performed in triplicate on three separate occasions, based on the method described by Searle *et al*. (2009) [23]. IPEC-J2 (passage 70–72) cells were seeded at 1.6×10^5^ cells/ml into 24 well plates and cultured using IPECs media consisting of: Dulbecco's Modified Eagle's Medium (DMEM; Sigma, UK) supplemented with 5% foetal bovine serum (Sigma, UK), 1% 2 mM l-glutamine (Sigma, UK), 1% sodium pyruvate (Sigma, UK) and 1% ITSS (Sigma, UK). Following 48 hours incubation at 37°C (100% confluence) the monolayers were washed three times with Hank's balanced salt solution (Sigma, UK). Each washed monolayer was inoculated separately with 1 ml of *S*. Derby D1 and D2, *S*. Mbandaka M1 and M2, *Escherichia coli* K12 DH5*α* (non-invading control) and a blank PBS control. This was performed in triplicate for each strain. For these studies, inocula were cultured to mid-log phase, standardised to an OD_540nm_ of 1.2 in PBS, and further diluted 1:20 in IPECs medium. Inoculated monolayers were incubated statically, at 37°C in 5% CO_2_. The assay was performed for three different incubation periods, 15, 30 and 60 minutes to identify variation in association and invasion rate between *S*. Derby D1 and D2 and *S*. Mbandaka M1 and M2. After incubation, monolayers were washed a further three times with Hank's balance salt solution. To the plates designated for studying invasion, 1 ml of IPECs medium containing 1% gentamicin (Sigma, UK) was added to each well, to kill external, but not internal, bacteria. Invasion plates were incubated for a further two hours at 37°C in 5% CO_2_. After incubation the monolayers were washed a further three times with Hank's balance salt solution (Sigma, UK).

Monolayers of both preparations were disrupted with individual, sterile, magnetic stirrers in parallel for 10 minutes in 1 ml of 1% TritonX (Sigma, UK) diluted in PBS. Serial dilutions between 10^0^–10^-8^ for each preparation including the initial standardized cultures were made in PBS, plated on to LB agar and incubated at 37°C for 16 hours. Colony forming units per ml were determined. T-tests were performed between the number of colony forming units per ml (CFU/ml) enumerated for all strains at each time point.

### Porcine *in vitro* organ culture and tissue association assay

*In vitro* organ culture (IVOC) was performed as previously described by Collins *et al.* [12]. On two separate occasions, three, six-week old, cross bred commercial pigs were stunned and euthanized through exsanguination. Jejunum and colon tissues were immediately removed from each pig and stored in separate Duran (Duran group, Germany) bottles containing 300 ml of pre-chilled IVOC medium which comprised RPMI 1640 medium (Sigma, UK) containing 10% foetal bovine serum (Sigma, UK), 0.25% lactalbumin hydrosylate (Sigma, UK), 75 mM mercaptoethanol (Sigma, UK), 0.2 µg ml^−1^ hydrocortisone (Sigma, UK) (1:1 chloroform/ethanol), 0.1 µg ml^−1^ ITSS (Sigma, UK), and 2 mM L-glutamine and L-aspartate (Sigma, UK). Jejunum and colon tissues were opened through a single transverse incision, followed by washing through submersion in PBS. The clean tissues were maintained in IVOC medium. Tissues were cut with a scalpel into 2 cm^2^ squares; sections were stored in IVOC medium until all tissues had been processed. Sections were mounted on CellCrowns (Scaffdex, Finland) with the mucosal side facing inwards, so as to form the base of the compartment. Crowns were placed into 24 well plates containing 1 ml of IVOC medium.

Association assays were performed on the mounted jejunum and colon tissues on two separate occasions. Mid-log cultures of *S*. Derby D1 and D2 were standardised in PBS to an OD_540nm_ of 1.2, PBS controls were also produced to determine bacterial contamination from the gut microflora, from these preparations a 1:20 dilution was made in IVOC medium pre-warmed to 37°C. Mounted tissues were inoculated in quadruplicate for RNA expression and association assays with 1 ml of each inoculum. A no tissue control (just bacteria) was also included for the RNA samples. Plates were subsequently incubated for 30 minutes at 37°C with 5% CO_2_. After incubation tissues were washed in 2 ml of sterile PBS; samples for RNA were added to 1 ml Tri-reagent (Sigma, UK), samples for enumeration were added to 10 ml of PBS.

Samples for enumeration were homogenised using a D-7801 hand held homogenizer equipped with emulsifier blades (Ystral, Germany). Homogenates were diluted in PBS in a series between 10^−1^ to 10^−4^ and were plated onto BGA (Oxoid, UK) supplemented with 1 µg/ml novobiocin (Sigma, UK) to enrich for *S. enterica* [24]. Plates were incubated for 16 hours at 37°C, prior to enumeration. T-tests were performed between the number of colony forming units per ml (CFU/ml) enumerated for all combinations of strains and tissues.

### RNA extraction and quantitative RT-PCR (qRT-PCR)

Colon and jejunum IVOC sections infected with *S*. Derby D1 and blank controls, as well as no tissue controls were stored in Tri-reagent at −80°C until RNA purification was performed. Samples were thawed on ice and homogenised using a Retsch MM301 bead homogeniser with 2.5 mm steel beads for 2 minutes at 30S^−1^ (Retsch, Germany). RNA was extracted using the Tri-reagent (Invitrogen, UK) chloroform method as per manufacturer's instructions. The final pellets were re-suspended in nuclease free water. Genomic DNA was removed using two treatments with DNA free (Ambion, UK) as per manufacturer's instructions and confirmed by the absence of a band after gel electrophoresis of a PCR reaction for the housekeeping gene *aroC* (primer sequences can be found in the **Table S1**). qRT-PCR reactions were performed using one-step Brilliant II SYBR Green with low ROX master mix (Stratagene, UK) as per manufacturer's instructions. In brief, 25 µl reactions were performed consisting of, 12.5 µl of Brilliant II SYBR green with low ROX master mix, 8.5 µl of nuclease free water, 1 µl of reverse transcriptase/RNase block enzyme, 1 µl of RNA (200 ng), and 1 µl (200 nM) of forward and reverse primers specific to the genes *gmk, sanA, potR, talN, chlR, genE, shaU, dumE, sadZ, tinY* and *docB* (primer sequences can be found in **Table S1**). For all primer sets and treatments, control reactions were performed, comprising either whole cell DNA extract of *S*. Derby D1, RNA with no reverse transcriptase or reverse transcriptase with no nucleotide template. Reactions were performed on a Stratagene Mx3000P, the parameters of the reaction were in accordance with the 2-step reaction recommended by Stratagene for the one-step Brilliant II SYBR Green with low ROX master mix with an annealing temperature of 52°C. The threshold cycle (CT) was calculated for each reaction by the Stratagene Mx3000P software V2.0 (Stratagene, UK) and standardised to the reading for the constitutively expressed guanylate kinase gene, *gmk*, as previously described [25], [26]. The efficiency of each PCR reaction was determined using dilutions of DNA template in nuclease free water to 1:10, 1:100, 1:1000; this was then used to adjust the relative concentration of each transcript. Results are given as fold changes relative to the readings for the no tissue control. T-tests were performed on the fold change values between jejunum and colon, p-values below 0.05 were deemed to be significant.

### Mutant construction and validation

The gene *potR* was deleted from the chromosome of *S*. Derby D1 using the Quick & Easy *E. coli* Gene Deletion Kit (Genebridges, Germany), as per manufacturer's instructions. In brief, a 10 ml overnight culture of *S*. Derby D1 was made electrocompetent through five successive rounds of centrifugation at 4000 rpm for 10 minutes at 4°C followed by re-suspension in ice cold distilled water and incubation on ice for 20 minutes. A final round of centrifugation was performed, this time the pellet was re-suspended in 200 µl of distilled water containing 10% glycerol. Cells were stored on ice until used.

To delete the gene *potR* from SPI-23, a kanamycin resistance cassette under a pGB2 promoter (Genebridges, Germany) was produced so that it was flanked by 50 bp of sequence homologous to the regions either side of the gene, as per manufacturer's instructions. In brief, the site specific homology arms were introduced into the cassette by designing 20 bp primers (Sigma, UK) for the 5′ and 3′ ends of a FRT-pPGK-pGB2-neo-FRT cassette (Genebridges, Germany) with an additional region of 50 bp that was homologous to either the 5′ or 3′ sequence adjacent to *potR* (primer sequences can be found in **Table S1**). PCR products were purified through gel electrophoresis, staining with ethidium bromide and were excised from the gel and purified using a QIAquick gel extraction kit (Qiagen, USA) as per manufacturer's instructions. The concentration of the purified cassette was determined using a Nanodrop N1000, spectrophotometer (Nanodrop, USA).

Electroporation was performed twice, once to introduce a plasmid carrying Red/ET recombinase under a temperature dependent promoter (Genebridges, Germany), and secondly to introduce the cassette with homology arms for site specific recombination with *potR*. In both cases, 2 µl of nuclease free water containing approximately 200 ng of DNA (either cassette or plasmid) was added to 40 µl of electrocompetent cells in a chilled 1 mm gap electroporation cuvette. Electroporation was performed with the following parameters: charging voltage 1.8 kV, resistance 600 ohms, pulse time of 4.2 m/s and capacitance timing 25 µF. Selection of, and recombination in, transformed strains was performed as per manufacturer's instructions (Genebridges, Germany). The correct insertion of the kanamycin cassette and deletion of *potR* was confirmed through colony PCR of the cassette chromosome margins and the central 1 kb of the gene (primer sequences can be found in **Table S1**). The mutant strain is from here on referred to as *S*. Derby D1 *ΔpotR*::kan.

### Negative staining of *S*. Derby D1 parent and *ΔpotR*::kan mutant

Negative staining was performed as follows, a 50 µl of planktonic bacterial suspension was air dried onto a glow discharged formvar/carbon coated support grid. Grids were then negatively stained with 2% phosphotungstic acid (pH 6.6) and immediately examined under a FEI Tecnai 12BT transmission electron microscope (FEI, USA) at 80 kV.

### Differential staining and confocal microscopy of invaded and associated bacteria to IPEC-J2 monolayers

IPEC-J2 monolayers were cultured on cover slips. The monolayers were exposed to *S*. Derby D1 and *S*. Derby D1 *ΔpotR*::kan as described above for the association assay. The monolayers were incubated with the inoculum for 4 hours to allow for a large number of bacteria to adhere and invade. After the incubation step, the inoculum was removed and the infected cells were fixed with 4% paraformaldehyde for 16 hours at 4°C, instead of being homogenised with 1% TritonX as in the above described association assays. Cover slips were removed from fixative and washed three times in PBS before being blotted with tissue paper and placed monolayer side up in a fresh 12 well plate. Goat anti-*Salmonella* O4 antibodies were added to the centre of each cover slip and incubated at room temperature under tin foil for 45 minutes, at which point the cover slips were removed from the well, washed three times in PBS and added to a fresh 12 well plate. This was repeated three times with the antibodies, donkey anti-goat labelled with Alexa 488 (Invitrogen, UK), goat anti-*Salmonella* O4, and donkey anti-goat labelled with Alexa 555 (Invitrogen, UK). Between the applications of antibodies Alexa 488 and the second application of goat anti-*Salmonella* O4, the IPEC-J2 cells were made permeable to antibodies through 10 minutes incubation with 0.3% TritonX diluted in PBS. After the final antibody was washed off the coverslips were dried with tissue paper and placed monolayer side down on to 5 µl drops of DAPI (Vector Laboratories, UK) on glass slides. The slides were incubated at room temperature under tin foil for 10 minutes, after which a thin layer of nail varnish was painted around the edge of the cover slips. Slides were viewed on a Leica TCS SP2 AOBS confocal system (Leica, Germany) attached to a Leica DM IRE2 microscope (Leica, Germany) equipped with ArKr laser excitation (488 nm), HeNe laser excitation (543 nm) and a diode laser (405 nm). Oil-immersion objective lenses (40× and 63×) were used, and imaging parameters were selected to optimise resolution.

### Characterising colony morphology and culture agglutination

*S*. Derby D1 parent and D1 *ΔpotR*::kan mutant were cultured from a frozen stock in 2 ml of pre-warmed LB at 37°C with agitation. From these cultures 100 µl was plated onto dried LB plates and incubated for 16 hours at 37°C. The remainder of the culture was left at room temperature for 2 hours before the cultures were gently agitated by hand and photographed.

### Bioinformatics

The conserved domains and homologous sequences of the amino acid sequence of *potR* were identified using BLASTp against a non-redundant protein database (using default settings, Accessed 5/12/13).

### Ethics statement

All studies involving the use of animals were reviewed and approved by the Animal Health and Veterinary Laboratories Agency (AHVLA) ethics committee. None of the research presented in this manuscripts was performed on live animals. For the IVOC experiments, six, six-week old, cross bred commercial pigs were electrically stunned and euthanized through exsanguination, a schedule 1 approved method, prior to post-mortem examination and tissue collection.

## Supporting Information

Table S1 **Primer sequences.** Sequences used in, the creation of the *potR* mutant, qRTPCR for SPI-23 expression profiling and to test for DNA contamination of DNAse treated RNA samples.
(XLS)
